# Supplementary material for: Copper-based grape pest management has impacted wine aroma
Source: Sci Rep. 2024 May 2;14:10124. doi: 10.1038/s41598-024-60335-9 (PMC11066116; doi:10.1038/s41598-024-60335-9)
Supplement: Supplementary file 1 — Supplementary Figures. [file 41598_2024_60335_MOESM1_ESM.docx]

**Supplementary Figure 1.** Variability of cumulate H_2_S production during alcoholic in strains coming from different ecological niches, in absence (**a**, in light yellow) or presence (**b**, in light red) of SO_2_. *Oak*: strains isolated from oak barks, *Velum*: yeast colonizing the wine velum in specific aging processes, *Wine*: strains isolated from wine fermentations. Means of each group for each condition are shown in Figure **1a** and **1b**.


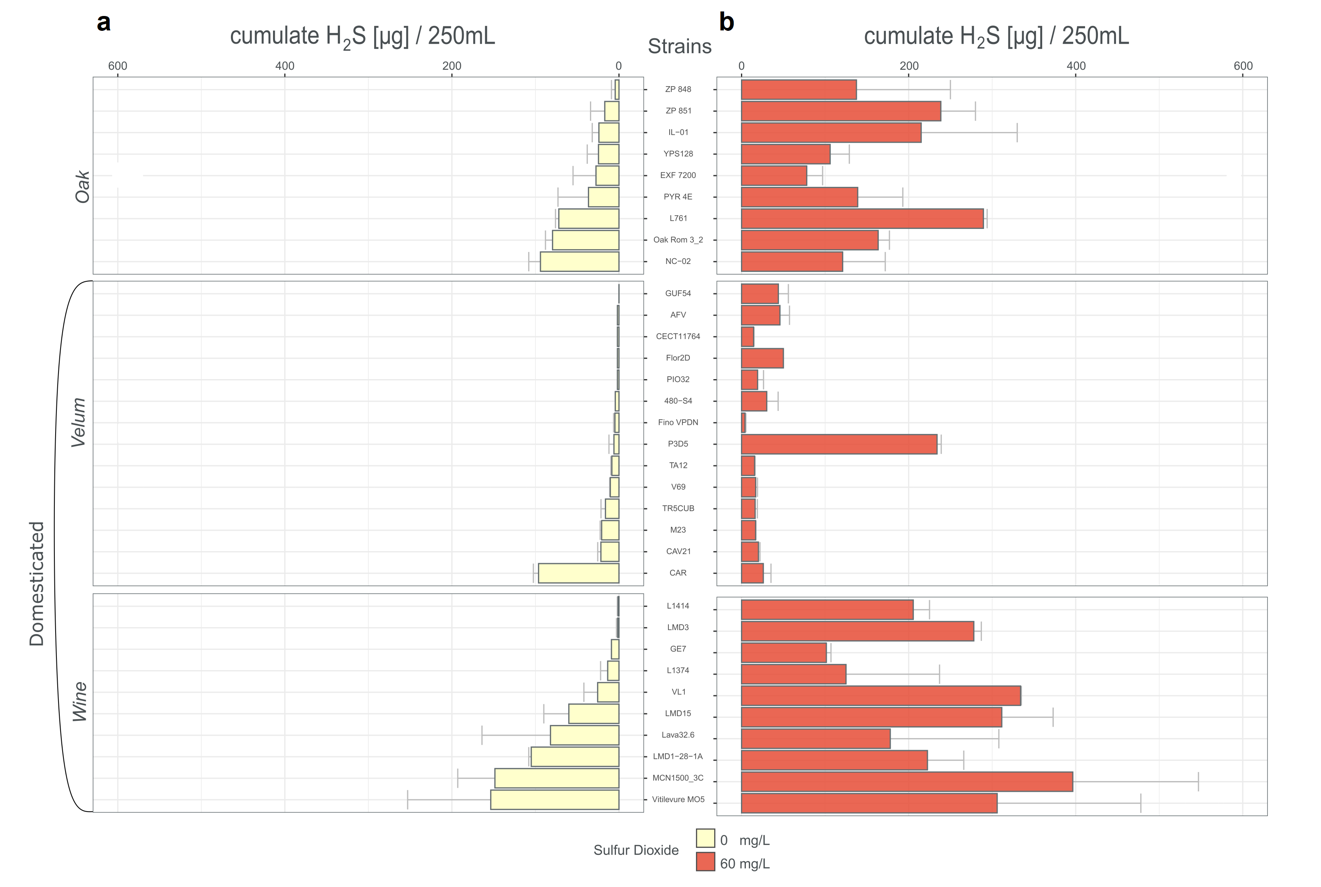


**Supplementary Figure 2.** Comparison of fermenters’ aspects at the end of alcoholic fermentation of synthetic must containing increasing amount of copper (0.25, 1, 2 and 5 mg/L), for wine yeast strain VL1 (A) and LMD17 (B). H_2_S productions of this experiment are shown in Figure 2.


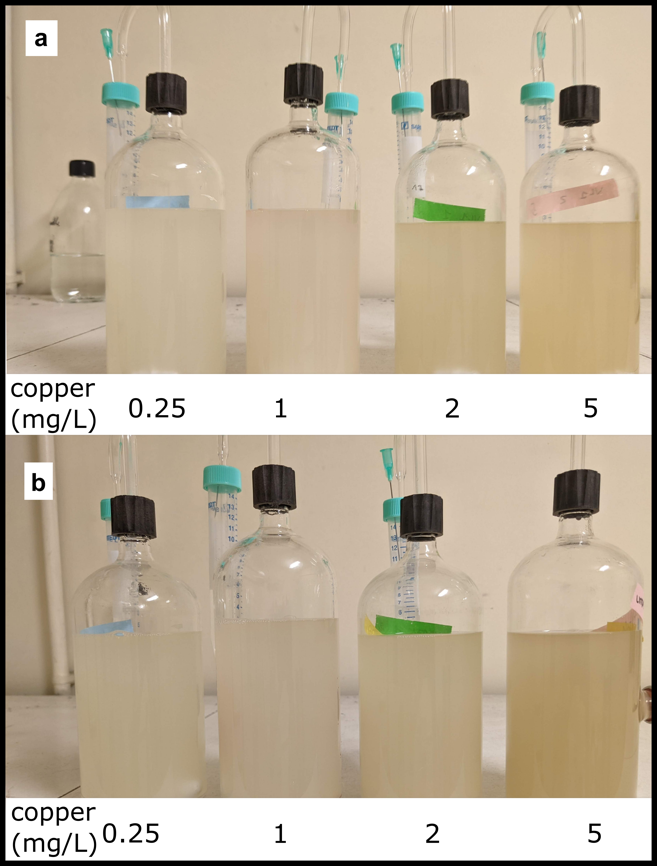


**Supplementary Figure 3.** Effect of the overexpression of CUP1 carried by a multicopy plasmid on Oakrom 3-2 growth at 28°C, in MS media containing geneticin (40mg/l).
